# Supplementary material for: Assessment of Genetic Variability and Bran Oil Characters of New Developed Restorer Lines of Rice (Oryza sativa L.)
Source: Genes (Basel). 2022 Mar 13;13(3):509. doi: 10.3390/genes13030509 (PMC8951205; doi:10.3390/genes13030509)
Supplement: Supplementary file 1 [file genes-13-00509-s001.zip › genes-1593447-supplementary.pdf]

**Table S1**: Analysis of variance for the grain yield, grain shape (L/B ratio), and amylose content percentage of the studied genotypes during 2019 and 2020 growing season.

| Traits | Mean Sum of Squares | | | | | | CV | |
|---|---|---|---|---|---|---|---|---|
| | 2019 | | | 2020 | | | | |
| | Replication | Genotype | Error | Replication | Genotype | Error | 2019 | 2020 |
| **d.f** | 2 | 4 | 8 | 2 | 4 | 8 | | |
| Grain yield per plant t/h | 0.63 | 6.24** | 0.62 | 0.25 | 8.66** | 0.40 | 6.79 | 5.12 |
| Grain shape | 0.004 | 0.028** | 0.002 | 0.017 | 0.077** | 0.004 | 1.77 | 2.36 |
| A.C% | 0.23 | 782.27** | 0.90 | 2.66 | 89.38** | 1.23 | 1.98 | 4.37 |

d.f: Degrees of freedom **: Highly significant at 1%

**Table S2.** Analysis of variance for chemical composition percentage of milled rice grains of the studied genotypes during the 2019 and 2020 growing season.

| **Chemical Composition** | **Mean Sum of Squares** | | | | | | CV | |
|---|---|---|---|---|---|---|---|---|
| | 2019 | | | 2020 | | | | |
| | Replication | Genotype | Error | Replication | Genotype | Error | 2019 | 2020 |
| d.f | 2 | 4 | 8 | 2 | 4 | 8 | | |
| Moisture | 0.268 | 0.935** | 0.118 | 0.1192 | 0.90** | 0.055 | 2.85 | 1.96 |
| Crude Protein | 0.04 | 0.265** | 0.025 | 0.10 | 0.24** | 0.03 | 2.00 | 2.14 |
| Ether Extract | 0.013 | 0.038** | 0.005 | 0.001 | 0.042** | 0.001 | 8.58 | 4.46 |
| Ash | 0.01 | 0.02** | 0.001 | 0.01 | 0.02** | 0.001 | 4.39 | 5.61 |
| Available arbohydrates | 0.41 | 0.84** | 0.11 | 0.10 | 0.81** | 0.07 | 0.37 | 0.28 |

**d.f: Degrees of freedom **: Highly significant at 1%**

**Table S3.** Analysis of variance for gross chemical composition (%) and minerals content (mg /100 g) of stabilized rice bran samples of the studied genotypes during the 2019 and 2020 growing season.

| **Chemical Composition** | **Mean sum of squares** | | | | | | CV | |
|---|---|---|---|---|---|---|---|---|
| | 2019 | | | 2020 | | | | |
| | Replication | Genotype | Error | Replication | Genotype | Error | 2019 | 2020 |
| **d.f** | 2 | 4 | 8 | 2 | 4 | 8 | | |
| Moisture | 0.0193 | 1.1868** | 0.0500 | 0.070 | 1.155** | 0.051 | 2.74 | 2.77 |
| Crude Protein | 0.05 | 1.10** | 0.03 | 0.12 | 1.04** | 0.04 | 1.06 | 1.11 |
| Ether Extract | 0.026 | 1.461** | 0.019 | 0.077 | 1.460** | 0.021 | 0.61 | 0.64 |
| Ash | 0.01 | 0.09** | 0.01 | 0.03 | 0.09** | 0.01 | 1.22 | 1.02 |
| Crude fiber | 0.06 | 0.37** | 0.04 | 0.07 | 0.37** | 0.05 | 1.26 | 1.44 |
| Available Carbohydrates | 0.88 | 2.765** | 0.113 | 0.29 | 2.780** | 0.106 | 0.92 | 0.89 |
| Phosphorus (P) | 60.0 | 10860.0** | 110.0 | 60.0 | 10860.0** | 110.0 | 1.11 | 1.13 |
| Potassium (K) | 140.00 | 10806.9** | 90.00 | 140.00 | 9989.10** | 90.00 | 1.11 | 1.12 |
| Magnesium (Mg) | 7.800 | 279.406** | 3.300 | 7.800 | 279.406** | 3.300 | 1.33 | 1.35 |
| Calcium (Ca) | 1.80 | 32.28** | 1.55 | 1.80 | 32.67** | 1.57 | 3.66 | 3.92 |
| Sodium (Na) | 0.04 | 1.24** | 0.02 | 0.05 | 2.19** | 0.03 | 2.21 | 2.56 |
| Manganese (Mn) | 0.02 | 0.24** | 0.01 | 0.01 | 0.23** | 0.01 | 1.74 | 2.21 |
| Zinc (Zn) | 0.002 | 0.56** | 0.01 | 0.01 | 0.20** | 0.01 | 2.95 | 3.11 |
| Iron (Fe) | 0.050 | 1.600** | 0.022 | 0.057 | 1.583** | 0.021 | 2.01 | 1.99 |
| Copper (Cu) | 0.005 | 0.028** | 0.002 | 0.005 | 0.018** | 0.002 | 4.92 | 5.91 |

**d.f: Degrees of freedom **: Highly significant at 1%**

**Table S4.** Analysis of variance for some physical, chemical properties, and fatty acids profile of rice bran oil from some rice genotypes (dry weight basis) of the studied genotypes during the 2019 and 2020 growing season.

| physical, chemical properties, and fatty acids | Mean Sum of Squares (df) | | | | | | CV | |
|---|---|---|---|---|---|---|---|---|
| | 2019 | | | 2020 | | | | |
| | Reps. | Genotype | Error | Reps. | Genotype | Error | 2019 | 2020 |
| d.f | 2 | 4 | 8 | 2 | 4 | 8 | | |
| Refractive index (25°C) | 0.00001 | 0.0002* | 0.0001 | 0.000005 | 0.0002* | 0.0001 | 0.50 | 0.52 |
| Specific gravity (25°C) | 0.000003 | 0.00014** | 0.000001 | 0.000004 | 0.00014** | 0.000001 | 0.11 | 0.12 |
| Acid value (%) | 0.008 | 0.407** | 0.005 | 0.001 | 0.408** | 0.0002 | 2.95 | 0.63 |
| Peroxide value (meq/kg oil) | 0.001 | 0.32** | 0.001 | 0.002 | 0.32** | 0.0005 | 1.41 | 1.36 |
| Iodin value (gI/100 g oil) | 0.017 | 23.26** | 0.01 | 0.015 | 23.26** | 0.006 | 0.07 | 0.07 |
| Saponification value (mg KOH/g oil) | 1.49 | 23.56** | 0.57 | 0.65 | 22.76** | 0.40 | 0.41 | 0.34 |
| Unsaponifiable matter (%) | 0.05 | 0.13** | 0.02 | 0.06 | 0.13** | 0.01 | 3.38 | 3.35 |
| Myristic C14:0 | 0.0049 | 0.04** | 0.002 | 0.001 | 0.03** | 0.0004 | 8.82 | 3.35 |
| Palmitic C16:0 | 0.06 | 0.50** | 0.02 | 0.08 | 0.41** | 0.03 | 0.77 | 0.92 |
| Palmitoleic C16:1 | 0.003 | 0.02** | 0.0008 | 0.001 | 0.02** | 0.0002 | 6.21 | 3.11 |
| Stearic C18:0 | 0.01 | 0.114** | 0.005 | 0.02 | 0.109** | 0.01 | 3.72 | 4.23 |
| Oleic C18:1 | 0.19 | 1.58** | 0.05 | 0.23 | 1.43** | 0.20 | 0.55 | 1.08 |
| Linoleic C18:2 | 0.04 | 1.80** | 0.05 | 0.56 | 1.97** | 0.20 | 0.67 | 1.33 |
| Linolenic C18:3 | 0.01 | 0.32** | 0.003 | 0.02 | 0.31** | 0.01 | 2.70 | 5.58 |
| Arachidic C20:0 | 0.008 | 0.083** | 0.002 | 0.001 | 0.082** | 0.0004 | 6.58 | 3.06 |
| Eicosenoic C20:1 | 0.001 | 0.0029** | 0.0003 | 0.001 | 0.0030** | 0.0003 | 1.66 | 1.95 |
| TSFA % | 1.21 | 2.67** | 0.300 | 1.19 | 2.09* | 0.301 | 2.32 | 2.49 |
| TUSFA % | 0.06 | 0.57** | 0.029 | 0.05 | 0.50** | 0.028 | 0.210 | 0.215 |

d.f: degrees of freedom **: Highly significant at 1% *: Significant at 5%
